# Comparison of sandblasted, ground and melt-etched zirconia crowns regarding adhesion strength to resin cement

**DOI:** 10.1080/23337931.2019.1621179

**Published:** 2019-06-05

**Authors:** Ketil Kvam, Aesha Irkayek, Eliza Vangaeva, Fadi El-Homsi

**Affiliations:** a NIOM – Nordic Institute of Dental Materials, Oslo, Norway;; b OsloMet – Oslo Metropolitan University, Oslo, Norway

**Keywords:** Zirconia surface treatments, tensile test, adhesion strength

## Abstract

**Objectives:** The aim is to compare the adhesion between zirconia and cements attained with melt-etching with potassium hydrogen difluoride, KHF_2_, with that found when such traditional surface treatments as sandblasting and ceramic stone grinding are employed.

**Materials and methods:** Groups of zirconia crowns where treated by sandblasting (*n* = 6), grinding with carbide bur (*n* = 6) or melt-etching with KHF_2_ (*n* = 6) of the surface before cementation with a resin cement to an implant substitute made by Selective Laser Melting of a cobalt-chromium alloy. Tensile testing was performed to rupture, while measured increasing load at the zirconia-cement interface. The strength was calculated by dividing the rupture load with the contact area. The three groups were compared using one-way ANOVA.

**Results:** The adhesion strength between the zirconia crowns and the cement resulted in significant differences between all groups (*p* < .05). The sandblasted group had the lowest strength (5.2 ± 0.95 MPa), the ground group significantly higher (7.3 ± 1.49 MPa) and the melt-etched group the highest values (9.8 ± 1.37 MPa).

**Conclusion:** The adhesive strength of resin cement to zirconia can be ranked according to the surface preparation with surfaces melt-etched with KHF_2_ stronger than ground which is stronger than sandblasted.

## Introduction

Since zirconia, i.e. zirconium dioxide based ceramics were introduced a few decades ago; it has become a common restoration material for crowns and bridges due to its high strength and the allegedly excellent biocompatibility [1]. An *in vivo* and *in vitro* study by Möller et al showed that zirconia-based implants had biocompatibility comparable with titanium implants [2]. Until recently the most common dental zirconia material has been yttria (Y_2_O_3_) stabilized tetragonal zirconia polycrystals with 3 vol% yttria (3Y-TZP) sintered at temperatures of 1300 °C to 1500 °C which maintain a stable tetragonal structure with a low quantity of cubic structure, 12.7–18.6 mass % respectively after cooling to usage temperatures [3]. A method to increase the translucency of zirconia increases the volume percent of yttrium oxides [4]. The fraction of cubic phase becomes higher than in the common 3Y-TZP in accordance to the phase diagram for zirconia-yttria [5]. Cubic zirconia is transparent; consequently a higher content of this phase results in greater translucency. However, the strength is reduced [6].

Bonding strength to different cements has been regarded as a factor limiting the use of zirconia materials. Good bonding requires a rough surface to enable mechanical bonding. Hydrofluoric acid etching is a common procedure for silica-containing glass ceramics, but the procedure is claimed not to give a surface on zirconia rough enough to improve the adhesion strength between cements and zirconia [7]. Another study had the same conclusion regarding the adhesion strength between cements and zirconia, but stated that a hydrofluoric acid treatment increased the structural transformation from tetragonal to monoclinic structure (t-m transformation) at the surface and reduced the flexural strength of zirconia, a phenomenon referred as ageing [8].

Different surface treatments of zirconia materials have been studied in recent years for their effect on bonding to a number of cements. Tribo-chemical silica coating was introduced by 3M ESPE AG in 1989 based on silicoating techniques for silicatization of metal frameworks. This method, the Rocatec system, has been used for both alumina- and zirconia-based ceramics [9]. The selective infiltration technique, SIE [10], SF_6_ plasma spraying [11] and laser irradiation [12–14] have also been attempted. The adhesion attained using these methods varied both before and after thermos-cycling in water. Some studies showed clear trends or predictions, but a review article [15] found no conclusive evidence for a universal treatment for clinically sufficient bonding using shear bond, tensile or micro-tensile tests.

A recently published article presented a melt-etching technique for zirconia surface treatment to enhance adhesion strength to resin cement [16]. The present study compares adhesion strengths for an inner zirconia surface of crowns which have been sandblasted, ground or melt-etched. The null-hypothesis was that no difference in adhesion strength could be attributed to surfaces treatment of the crown’s inner surface.

## Materials and methods

The test method uses a modification of the equipment designed for a pull-off test performed on alumina and zirconia crowns mounted to steel models by Derand et al. [17].

**Figure 1.**
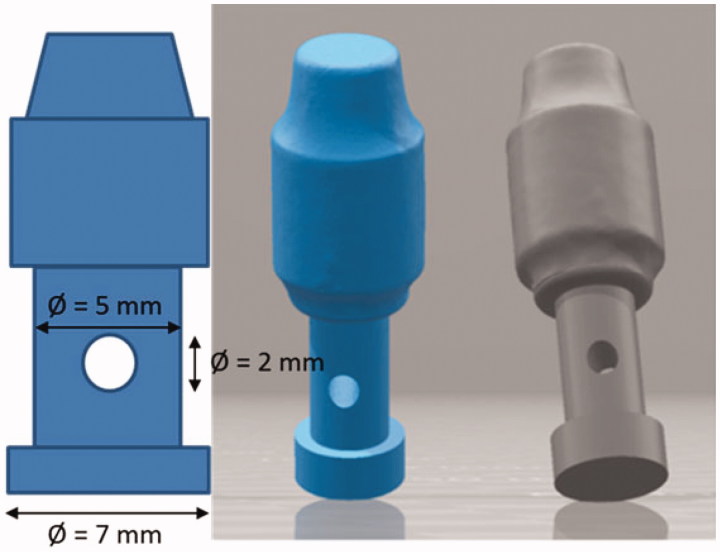
Dimensions and 3D view of the cobalt-chromium implant substitutes.

An implant substitute was designed by a 3D builder program (3D Builder, Microsoft, Dublin, Irland). One STL-file of this design was sent for production of 18 standardized implant substitutes in a cobalt-chromium alloy, Remanium^®^ star CL (Table 1), with Selective Laser Melting (SLM) by 3D-TEC Sweden AB (Kristianstad, Sweden) according to the design modifications (Figure 1).

**Table 1.** Materials used in the experiments.

Material	Producer	City, Country	Batch-nr.
Prettau Zirkonia (Y-TZP)	Zirkonzahn	Gais, Italy	ZB7287N
remanium^®^ star CL (CoCr-alloy)	Concept Laser GmbH	Lichtenfels, Germany	460121A
Potassium hydrogen difluoride, KHF_2_, Purum p.a.	Honeywell	Seelze, Germany	H054A
Multilink Hybrid Abutment	Ivoclar Vivadent AG	Schaan, Liechtenstein	V17296

**Figure 2.**
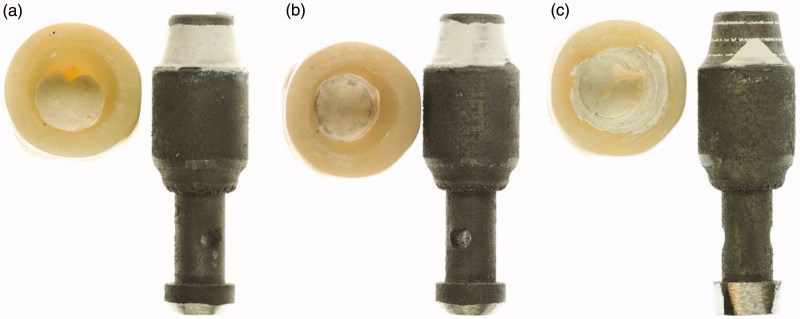
Crowns de-bonded from implant substitutes when the insides of the crowns are (a) sandblasted, (b) ground and (c) melt-etched with potassium hydrogen difluoride.

A model of tooth 36 was designed with CAD/CAM Zirkonzahn Modellier-system for making 18 standardized Prettau Zirconia crowns (Table 1) for mounting to the implant abutments (Figure 2). The crown model was designed for a cement gap of 30 µm and with a 2 mm wide cervical margin which enable a steel grip to be loaded in tension. An abutment and the crown model were scanned with Zirkonzahn scanner (Scanner S600 ARTI, Zirkonzahn, Gais, Italy) and 18 crowns were milled using a Zirkonzahn Milling machine M1 (Zirkonzahn, Gais, Italy).

The abutment substitutes were sandblasted with 110 µm alumina at an angle of 45° and a distance of 10 mm leaving circular traces as shown in Figure 2. This was done to ensure that the cement would adhere to the abutments and ensure that bonding between the cement and the zirconia surfaces on the inside of the crowns would be tested.

**Figure 3.**
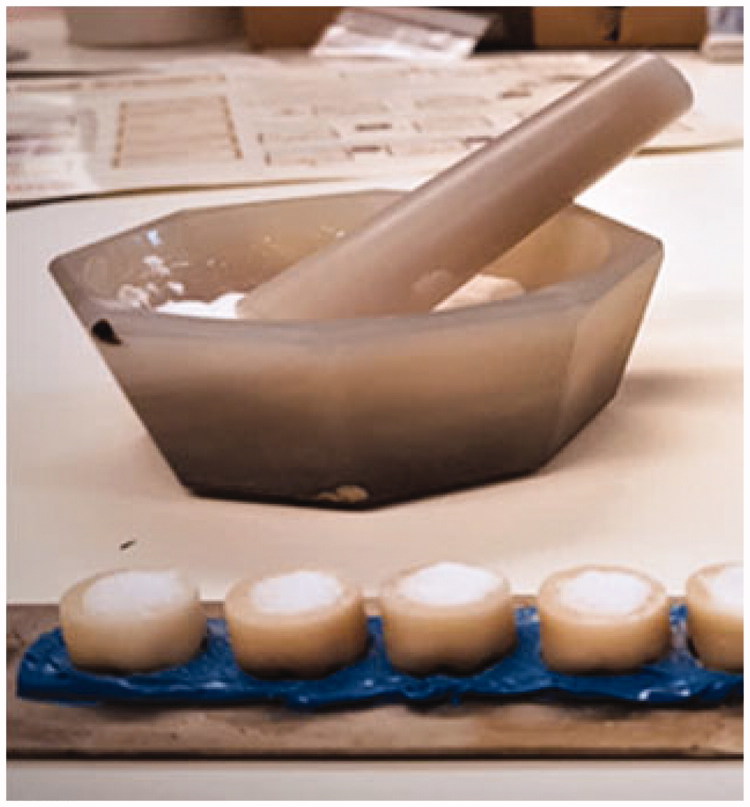
Potassium hydrogen difluoride crushed in a mortar and filled in crowns before melt-etching.

**Figure 4.**
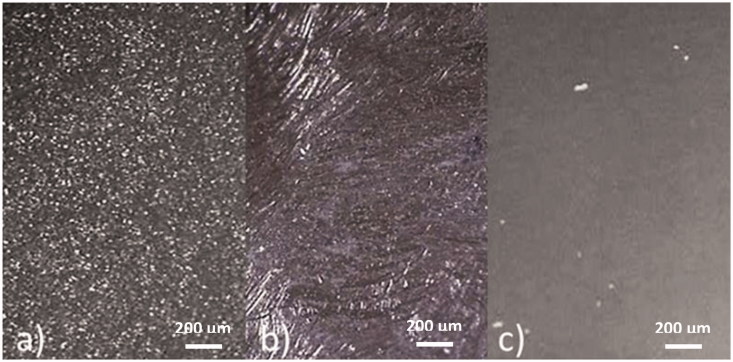
Inner surfaces of crowns, visualized in stereo microscope after (a) sandblasting, (b) grinding and (c) melt etching.

Three groups of zirconia crowns were given different inner surface preparations, A: sandblasted (*n* = 6), B: ground (*n* = 6) and C: etched (*n* = 6) as preparation for mounting to the cobalt-chromium implant substitutes. Group A was sandblasted for 10 s with 50 µm alumina particles at a pressure of 3.5 bars and at 45° angle to the inner surface. Group B was carefully hand milled at the inner side with a ceramic grinder tool (Diagen-Turbo-Grinder dtg ceramic (Bredent GmbH & Co, Senden, Germany). The crowns in Group C were filled with fine grained potassium hydrogen difluoride, KHF_2_ (Table 1), which had been crushed in an agate mortar (Figure 3). Etching was performed by heating in a furnace for 10 min at 270 °C. All crowns were thoroughly steam cleaned and ultrasonically rinsed in distilled water to ensure that no remnants of the treatments were left at the inner surfaces. In order to evaluate the fracture morphology, the inner surfaces of the crowns were visually inspected in a stereo microscope (Wild Photomacroscope M400, Wild Leitz, Heerbrugg, Switzerland) for evaluation of surface morphology after surface treatment both before and after the de-bonding test (Figure 4).

The crowns were mounted on the abutment substitute with a self-curing, resin based cement, Multilink Hybrid Abutment, recommended for zirconia (Table 1). To ensure equal pressure during setting of the cement, a controlled load of 50 N was applied for 5 min. The excess cement was then removed. Thereafter the crowns were stored in deionized water at 37 °C for 24 h before testing.

**Figure 5.**
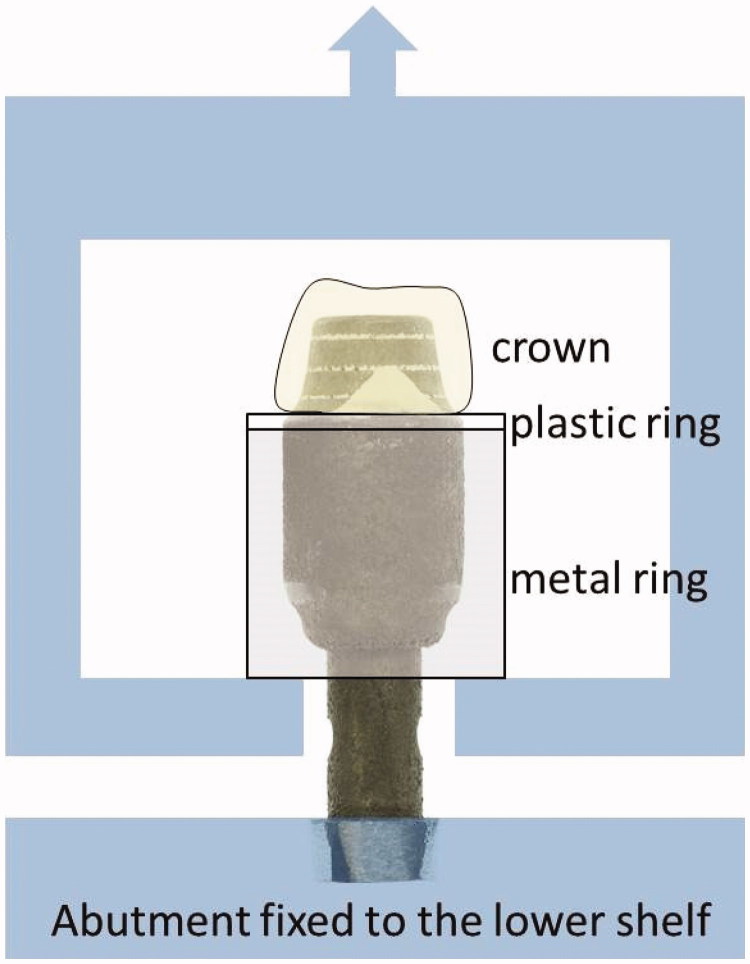
The tensile system schematically drawn with one of the group 3C abutment substitutes.

The abutment substitutes with the cemented crowns were tested in a universal test machine, Lloyds LRX (Lloyds Instruments Ltd, Fareham, UK). A metal cylinder and a plastic ring with an inner diameter less than the crown margin were placed around the abutment substitutes before these were mounted in the machine and clamped (Figure 5). The metal cylinder was then fixed to the upper drive to be pulled up during loading so that the plastic ring distributed the load around the crown margin. The crosshead speed was 1 mm/min. The tensile force was measured until the cemented crowns fully de-bonded from the abutments.

The adhesion strength, *σ*, was calculated as *σ* = *F*/*A*, where *F* was the force at rupture and *A*, the cemented area, calculated as a sum of the top circular area and a truncated cone with the upper radius of 3.2 mm and the margin radius of 4.0 mm and a height of 5.0 mm, i.e. 145.3 mm^2^ which is the same for all samples.

The de-bonding strength data was analyzed using One Way Anova followed by the Tukey’s multiple comparisons test using Graphpad, version 6.0 with a *p* values <.05.

## Results

When inspected in stereo microscope prior to cementation, the inner surface exhibited different morphologies according to group (Figure 4). The sandblasted group had a rough surface, the ground surface was also rough with grinding grooves in different directions and the melt-etched surface looked shiny, but was matte at high magnifications. After tensile testing, the fracture morphology showed combinations of adhesive fracture to zirconia and cobalt-chromium surfaces, and cohesive fractures in the cement (Figure 2).

The adhesion strength between the crowns and the abutments by tensile testing showed significant differences between the groups (*p* < .05). The sandblasted group had the lowest strength values and the melt-etched group showed the highest values (Table 2).

**Table 2.** Results of tensile testing at rupture, mean values and standard deviations.

Group	*F*_fail_ (N)	Bond strength (MPa)	Standard deviation (MPa)
Sandblasted	751	5.2	0.95
Ground	1067	7.3	1.49
Etched	1426	9.8	1.37

## Discussion

The aim of this study was to trial the etching method introduced by Ruyter et al. [16] on milled crowns made by a commercial dental CAD/CAM milling system and to compare this with surface treatments which are recommended by manufacturer of dental zirconia materials. For this limited study, only one of the cements recommended for zirconia materials was used. Other cement types may give a different result from the current study. However, the focus has been on the zirconia surface texture.

CoCr-alloy was chosen for the implant substitute material in this study. It would have been more natural to choose titanium or zirconia as the implant material, but the present study was focused on the bond strength between the zirconia crown material and the cement.

In a stereo microscope at low magnification (32X), only the sandblasted and ground surfaces appeared rough; the melt-etched surface looked smooth and shiny. At the higher magnification (64X), however, the melt-etched surface appeared to be matte indicating some roughness, apparently sufficient to provide a good retention. Static images of the texture of the inner curved surface taken at high magnification contained little information. However, Ruyter et al. [16], using scanning electron microscope on planar specimens, observed sub-micrometer scale undercuts due to melt-etching and 5 µm scale undercuts due to sandblasting. The melt-etching may also have left a surface with a surface energy that differed from the mechanical treatments and a better affinity to the resin cement. As discussed in a recently published study [18], the melt etch technique with KHF_2_ can have resulted in fluoridated surface which has attached hydroxyl groups after water cleaning giving a positive effect on the adhesion.

Sandblasting and grinding of zirconia surfaces are associated with an energy-induced structural transformation from tetragonal to monoclinic structure which is believed to enhance hydrothermal aging when exposed to dynamic loading in humid environment as described by several authors, including Prado et al. [19]. The melt etch method gives less structural transformation [16] and while, according to the present study, improving the adhesion to resin cement.

Despite claims that etching of zirconia is difficult and has a limited effect, the results of this present study show that melt-etching with potassium hydrogen difluoride has given significantly higher bond strength for resin-based cement than both sandblasted and ground surfaces. This confirms that the etching method proposed by Ruyter et al. can be successful when used on clinically designed crowns. The null-hypothesis can be rejected.

## Conclusions

There were significant differences between adhesion strength to resin cement for the sandblasted, ground and melt etched groups, with the melt etch method showing the highest strength.
